# Encoding information in synthetic metabolomes

**DOI:** 10.1371/journal.pone.0217364

**Published:** 2019-07-03

**Authors:** Eamonn Kennedy, Christopher E. Arcadia, Joseph Geiser, Peter M. Weber, Christopher Rose, Brenda M. Rubenstein, Jacob K. Rosenstein

**Affiliations:** 1 School of Engineering, Brown University, Providence, RI, United States of America; 2 Department of Chemistry, Brown University, Providence, RI, United States of America; University of Lincoln, UNITED KINGDOM

## Abstract

Biomolecular information systems offer exciting potential advantages and opportunities to complement conventional semiconductor technologies. Much attention has been paid to information-encoding polymers, but small molecules also play important roles in biochemical information systems. Downstream from DNA, the metabolome is an information-rich molecular system with diverse chemical dimensions which could be harnessed for information storage and processing. As a proof of principle of small-molecule postgenomic data storage, here we demonstrate a workflow for representing abstract data in synthetic mixtures of metabolites. Our approach leverages robotic liquid handling for writing digital information into chemical mixtures, and mass spectrometry for extracting the data. We present several kilobyte-scale image datasets stored in synthetic metabolomes, which can be decoded with accuracy exceeding 99% using multi-mass logistic regression. Cumulatively, >100,000 bits of digital image data was written into metabolomes. These early demonstrations provide insight into some of the benefits and limitations of small-molecule chemical information systems.

## Introduction

The metabolome is the complete set of small molecules found in a biological system [1]. The properties of this set of compounds are an amplified and dynamic measure of an organism’s genome, transcriptome, proteome, and environment [2]. This makes the metabolome an incredibly information-rich system, which displays diverse chemical, structural and biological dimensions [3–5].

Although much remains to be understood, improvements in protocols and efficient mass spectrometry (MS) have enabled metabolomic disease screening and drug discovery [6–12]. These technologies are supported by continually improving statistical tools and databases [13, 14]. As these tools advance, they may also suggest exciting alternative applications for metabolomics.

For inspiration, we observe that researchers have mimicked living systems by using DNA [15] for long-term archival information storage [16, 17], building on rapid advances in sequencing technology. Given recent progress in proteomic and metabolic profiling tools [18–21], it is timely to explore if the metabolome can also be used in a complementary way for information representations.

Whereas DNA and proteins are often large molecules which exist in small numbers, metabolites are higher in number, smaller in mass, and more structurally and energetically diverse. Like DNA, metabolites are biologically ubiquitous, and their primary pathways and processes are conserved across species [22]. The power of DNA as an information carrier comes from the combinatorial complexity that can exist within one polymer [23]. By contrast, the power of the metabolome is in the diversity of many co-existing molecules which can interact, or be acted upon, in complex combinations [5].

Non-genomic molecular data storage has also been demonstrated using fluorescent dyes on polymer films [24] and rotaxanes [25]. Other demonstrations have utilized collections of fluorophores which interact with information-bearing compounds in statistically identifiable ways [26]. However, all of these methods encode information into the state of a single compound at one time.

**Fig 1:**
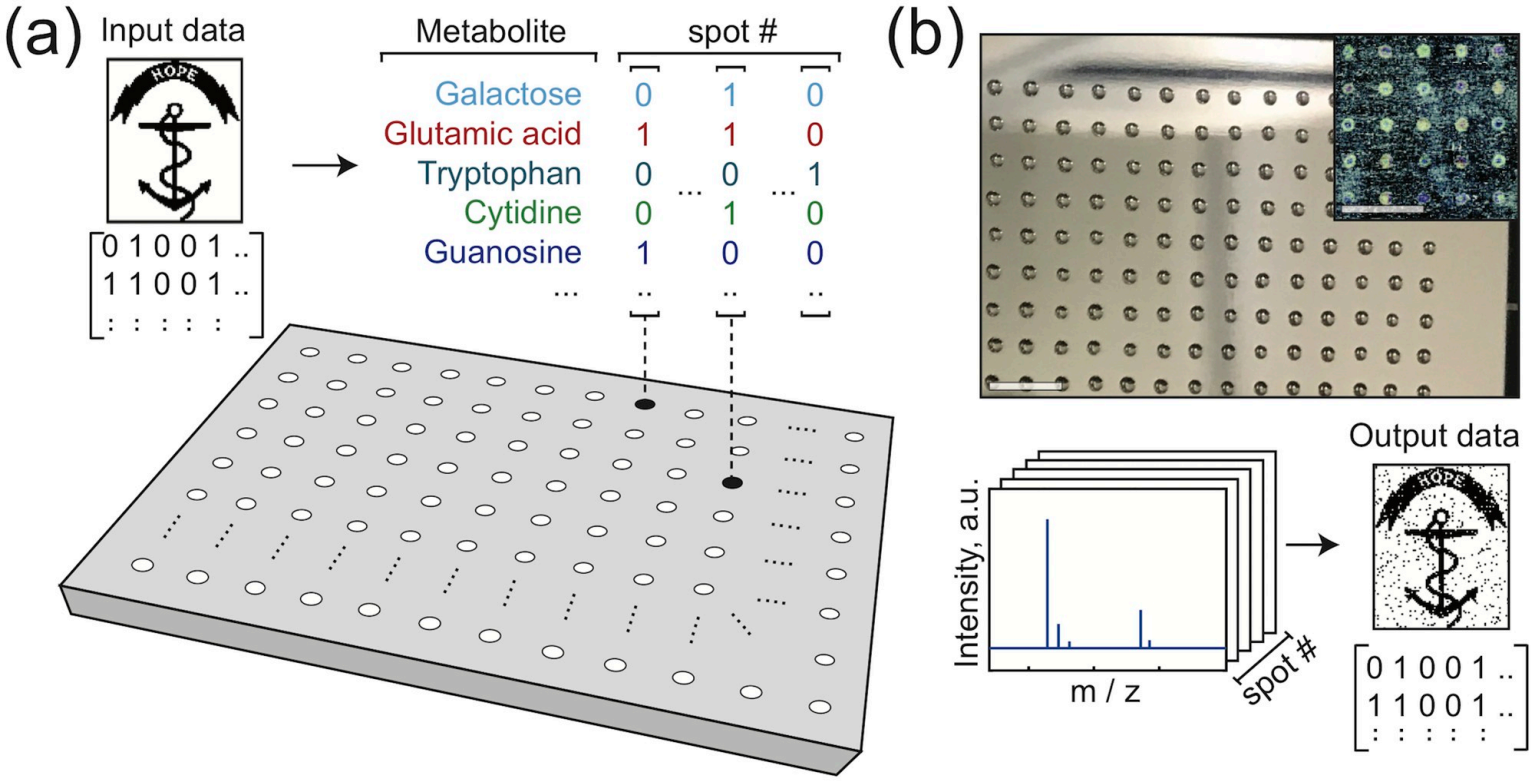
Writing and reading data encoded in mixtures of metabolites. (a) Binary image data is mapped onto a set of metabolite mixtures, with each bit determining the presence/absence of one compound in one mixture. For example, a spot mapped to four bits with values [0 1 0 1] may contain the 2^*nd*^ and 4^*th*^ metabolite at that location. **(b)** Small volumes of the mixtures are spotted onto a steel plate and the solvent is evaporated (scale bars: 5 mm). This chemical dataset is analyzed by MALDI mass spectrometry (b, bottom). Using the observed mass spectrum peaks, decisions are made about which metabolites are present. These decisions are assembled from the array of spots to recover the original image. The image shown is the Rhode Island Hope Regiment Colors [28].

In this paper, we encode abstract binary data into the chemical composition of thousands of spatially arrayed nanoliter volumes (Fig 1a). Each volume (‘spot’) contains a prescribed mixture from a library of purified metabolites—a synthetic metabolome. A key strength of this work is that it can be applied to any chemical library. Metabolites hold particular potential, because they provide access to well-regulated interconversion networks, materials, and databases which could facilitate computational operations on chemical data. The presence or absence of one metabolite in one spot encodes one bit of information. Therefore, the total number of bits stored in one spot is equal to the number of available library elements [27].

We recover the encoded data from metabolic mixtures using mass spectrometry (Fig 1b). The data aquisition is inherently parallelized, because a single mass spectrum provides information on every compound in a mixture. Noise characterization and logistic regression strategies for recovering the data are presented, along with examples of chemically encoded digital images. Raw error rates <1% are achieved with kilobyte-scale data sets using a simple peak analysis, illustrating the viability of both writing and reading metabolomic information. We use these experimental demonstrations to consider the benefits and limitations of encoding data into a biochemical medium in which interactions and interconversions can occur.

## Materials and methods

### Chemical library preparation

Reagent grade samples of 36 distinct metabolic compounds (Table A in S1 File) were diluted in dimethyl sulfoxide (DMSO, anhydrous), each to a nominal concentration of 25mM. Some metabolites were initially dissolved in an alternative solvent (de-ionized water with or without 0.5M or 1M hydrochloric acid) to facilitate solvation in DMSO. 10*μL* of each compound was aliquoted into a 384-well microplate (Labcyte 384LDV).

### Data mixture preparation

The chemical data mixtures were prepared on a 76 × 120 mm^2^ stainless steel MALDI plate. An acoustic liquid handler (Labcyte Echo 550) was employed to transfer the compounds from the library wellplate onto the MALDI plate. The nominal droplet transfer volume is 2.5 nL, but to reduce variability, we typically use 2 droplets (5 nL) per compound. The destinations of the droplets are programmed to match a standard 2.25mm pitch 1536-spot (32 × 48) target.

After spotting the compounds to the MALDI plate, a MALDI matrix material was added to each location. We selected 9-Aminoacridine for its compatibility with metabolite libraries, its low background in the small molecule regime, and its support for both positive and negative ion modes. The MALDI plate is left to dry and crystallize overnight (∼ 10 hours). Once dried, the plate can be stored in a humidity controlled cabinet or analyzed by MALDI-FT-ICR mass spectrometry.

### Mass analysis of data plates

A Fourier-transform ion cyclotron resonance (FT-ICR) mass spectrometer (SolariX 7T, Bruker) was used to analyze the crystallized metabolite data mixtures. The exact resolution is a function of the measurement time allocated per spectrum. For these experiments, we typically used 0.5-1 sec, yielding a resolution of < 0.001 Da. The instrument is run in MALDI mode and is configured to serially measure the mass spectrum of each mixture on the 48x32 grid. Acquisition for a full plate takes <2 hours.

To read the encoded data from the mass spectra, the probability of a metabolite being present is modeled as a combination of multiple predictor masses. A multinomial logistic regression considers the natural exponent of an offset plus the sum of all identifying mass SNRs, where each SNR is multiplied by a trained weight coefficient. A limited-memory BFGS algorithm was used to predict the logistic accuracy scores given an input of the *n* best peaks per metabolite. This process was iterated for all metabolome constituents.

## Results

### Writing synthetic metabolomes

Our synthetic metabolome is a diverse set of 36 compounds (Table A in S1 File) including vitamins, nucleosides, nucleotides, amino acids, sugars, and metabolic pathway intermediates (all purchased from Sigma-Aldrich). To write data into mixtures of metabolites, we use an acoustic liquid handler (Echo 550, Labcyte) to transfer pure metabolic solutions in 2.5 nL increments to pre-defined locations on a steel MALDI plate. A 2.25 mm pitch grid was chosen for compatibility with standard wellplate protocols. This produces a spatial array of different mixtures of metabolites where the presence (or absence) of each compound in each mixture encodes one bit of information.

After evaporating the solvent, each data plate contains up to 1536 dried spots (Fig 1b), which we can analyze using Matrix Assisted Laser Desorption Ionization (MALDI) mass spectrometry (MS). To prescreen each compound in the synthetic metabolome, a plate was written with combinatorial mixtures of all 36 metabolites across 1400 unique spots (Fig A in S1 File). Since MALDI protocols are chemically specific, we do not expect the same identification accuracy across the whole compound library under one set of conditions. We use this pre-screen to determine the MS identification accuracy for every metabolite with the same protocol.

### Ion cyclotron mass spectrometry of metabolite mixtures

A Fourier-transform ion cyclotron resonance (FT-ICR) mass spectrometer (SolariX 7T, Bruker) was used to analyze the array of crystallized mixtures. In FT-ICR MS, a pulsed RF field excites ions into a periodic orbit with a frequency that is determined by the magnetic field strength and the ion’s mass [29], which enables much finer mass resolution than time-of-flight (ToF) instruments. In these experiments, the mass resolution is typically 0.001 Da (Fig B in S1 File). Using FT-ICR MS, metabolites can be discriminated even if their masses are only milli-Daltons apart.

**Fig 2:**
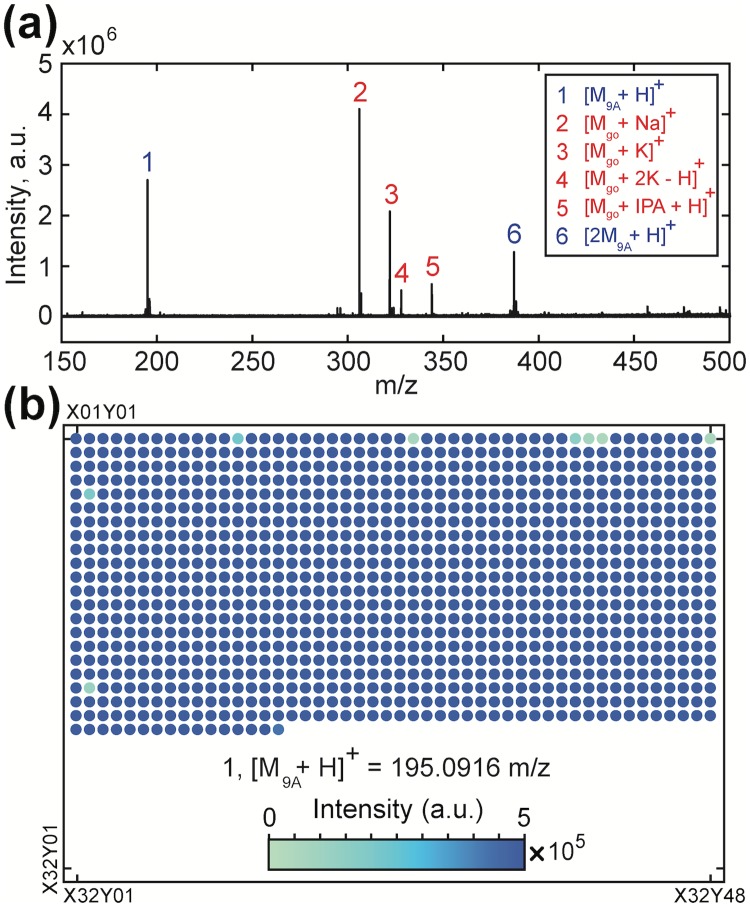
Analyzing chemical data plates with mass spectrometry. **(a)** Positive mode MALDI-FT-ICR mass spectrum of one spot containing Guanosine (*go*) and the MALDI matrix, 9-Aminoacridine (*9A*). Automated analysis of each spot used 4x averaged 1-second acquisitions. *go* ions (2, 3, 4, 5, in red) are present, along with two protonated matrix peaks (1, 6, in blue). **(b)** The intensity of the protonated matrix (peak 1) at m/z = 195.0916 ± 0.001 is shown graphically for a MALDI plate with 1024 independent mixture spots. Protonated aminoacridine is positively identified in 1020 spots (99.6%).

In Fig 2(a), one positive-ion MALDI-FT-ICR mass spectrum is shown for a spot which included guanosine (*go*), together with 9-Aminoacridine (*9A*) matrix. Protonated matrix adducts are identified at peaks 1 and 6 (blue), along with adducts of guanosine, labeled (2: Na, 3: K, 4: 2K—H and 5: isoproyl alcohol (IPA) + H). The observed intensities vary by adduct and species. In Fig 2(b), the intensity of the first peak (protonated matrix at m/z = 195.0916 ± 0.001) is illustrated across 1024 spots.

Many open-access tools are available for metabolite peak detection and assignment from MS spectra [21]. To clearly relate the mass spectra to binary data, we consider a rudimentary detection scheme: if a metabolite’s mass intensity is above a particular threshold, then it is declared present, and the binary state of its address is set to 1 (or to 0, if its mass peak is absent). This approach identified the substrate matrix protonated peak in 1020 out of 1024 spots (≈ 99.6%) in Fig 2(b).

As an inital demonstration, we selected a library subset of 6 metabolites, which were used to encode a 6,142-pixel binary image of a Nubian ibex [30] into an array of 1024 mixtures (Fig C in S1 File). After pseudo-random interleaving, the data was mapped onto the presence or absence of sorbitol (*so*), glutamic acid (*ga*), tryptophan (*tp*), cytidine (*cd*), guanosine (*go*) and 2-deoxyguanosine hydrate (*gh*). The plate was written and then analyzed using FT-ICR MS as detailed in the Methods.

**Fig 3:**
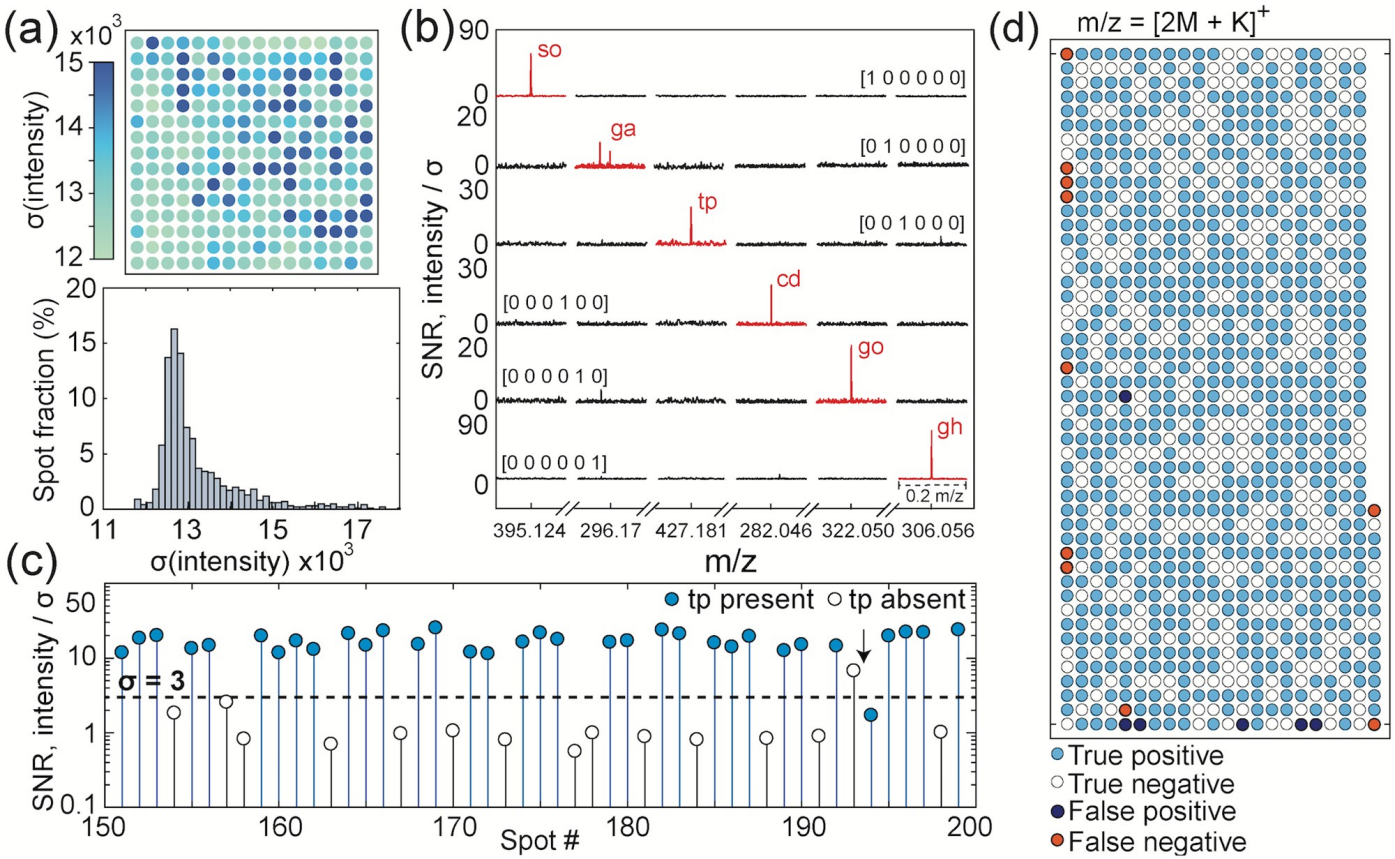
Spectral background and noise considerations. **(a)** Heatmap of the standard deviation of the MALDI-FT-ICR-MS spectral background noise from 240 unique spots of distinct mixtures, and a histogram of the background intensity standard deviation. **(b)** Spectra for six metabolites, normalized by the noise standard deviation. The m/z is cropped to six ranges of interest. Six ‘one-hit’ mixtures are plotted, one for each metabolite. **(c)** To assign presence/absence, we choose an intensity threshold at an appropriate m/z. As shown here, a 3*σ* threshold applied to the [2M_*tp*_ + K]^+^ tryptophan peak yielded a discrimination accuracy of 96%. **(d)** A hit map of the same tryptophan peak illustrating recovery using the 3*σ* threshold. Interestingly, the few errors are clustered at the edges of the plate.

Fig 3a presents a spatial map and histogram of the spectral background noise observed in 240 independent spots. Before further analysis, we divide each spectrum by its background *σ*, which allows more direct comparison of signal strength at multiple locations. Signal strength is a complex function of the sample preparation, analyte and adduct. After normalization, peaks of interest for the 6 metabolites are shown in Fig 3b. The first row is a spot whose data contains the six bits [1 0 0 0 0 0], and thus only the m/z peak associated with the first metabolite (sorbitol) is present. Similarly, five other ‘one-shot’ patterns are shown which can be decoded without error.

A threshold of 3*σ* was chosen as the intensity required to declare the presence of a metabolite. For example, if we examine the tryptophan [2M_*tp*_ + K]^+^ mass (Fig 3c), we find that this threshold yields 96% correct classification. This detection scheme can also be visualized for each spot on the plate, as shown in Fig 3d. Clustering of errors at the edges of the plate suggests that small misalignments between the MALDI laser positions and the droplet spotting locations were a source of error.

### Statistical analysis of data plates

**Fig 4:**
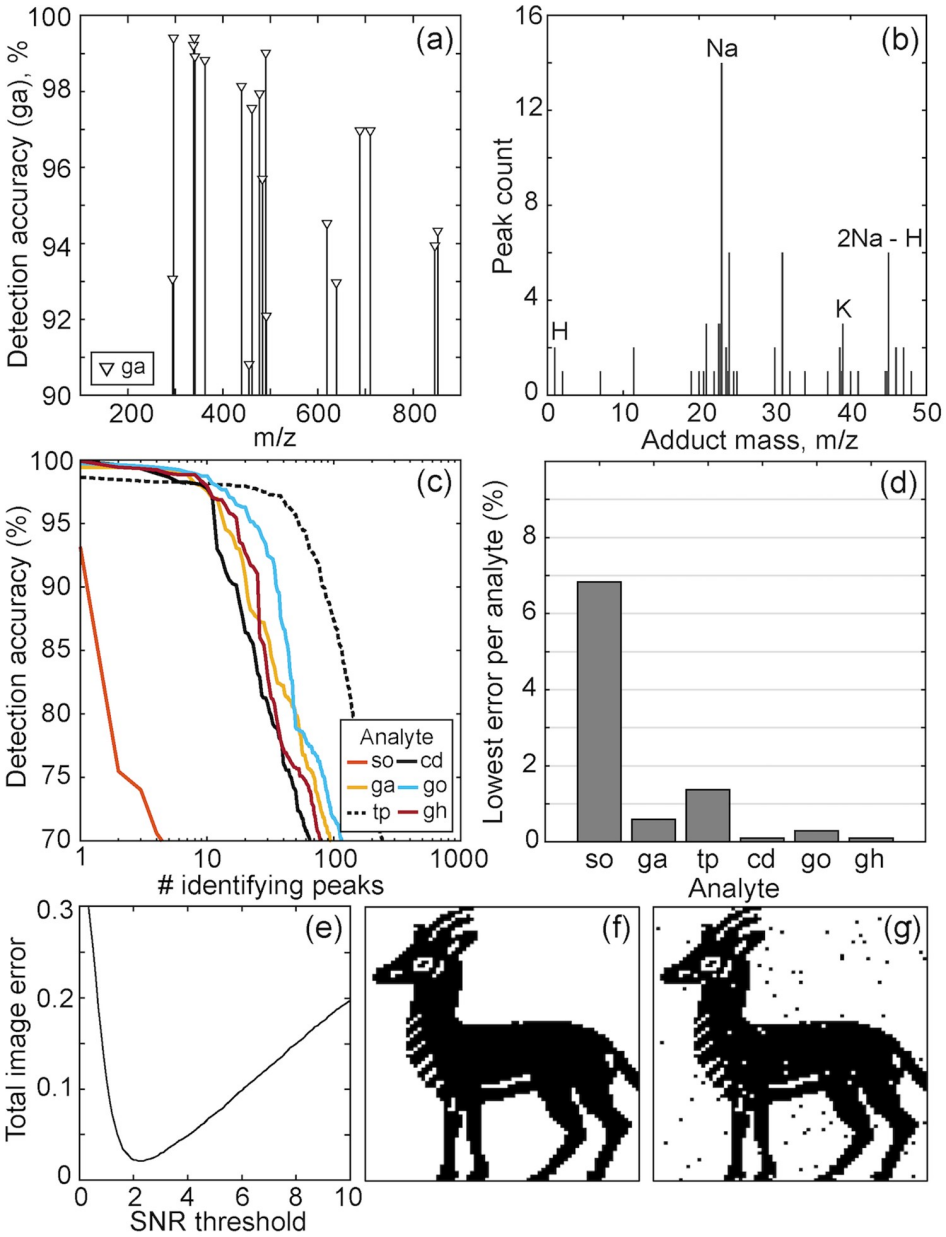
Identifying discriminating peaks. **(a)** The read recovery for different masses across the MS spectrum is shown for *ga*. **(b)** The histogram of adducts associated with peaks from the data in Fig 3 indicates sodiated ions are predominant. **(c)** For each metabolite, we plot the number of peaks achieving a given detection accuracy. With the exception of sorbitol, each metabolite has at least 10 identifying peaks with >97% accuracy. **(d)** The error of the single best performing mass for each metabolite. **(e)** Using only the best performing mass from (d), the error rate for the six metabolites across 1024 locations (6144 bits) is shown as a function of the SNR cutoff. These mixtures encoded the 6142-bit image shown in **(f)**. In **(g)**, we recover the image with a 2.5*σ* decision threshold, producing approximately 2% cumulative read/write error.

In practice, one compound will be associated with multiple peaks, having varying signal-to-noise ratios and usefulness. For a given metabolome, we should attempt to identify which m/z peaks are most appropriate to identify each library element. Each high-resolution FT-ICR mass spectrum contains ∼2 × 10^6^ m/z points. Since most of the spectral space is background, it is helpful to first reduce the number of features to those which may be statistically useful. 1,444 candidate peaks found in the ensemble average of all mass spectra were tested to determine how accurately the intensities at that m/z classified the encoded data values (Fig 4a).

Although these peaks were identified without chemical bias, many features can be attributed to known metabolite adduct ions (although some are synthesis byproducts or derive from the substrate matrix). A histogram of the associated adduct masses is shown in Fig 4b. H, Na, Na-H and K adducts are all frequently observed.

The number of peaks achieving detection accuracy in the range of 70-100% is shown in Fig 4c. Selecting the best performing peak for each metabolite, and applying a detection threshold of 2.5*σ*, was sufficient to recover data at about 2% cumulative read/write error (Fig 4e). The corresponding input and output data images are shown in Fig 4f and 4g. The simplicity and success of the overall read and write process is encouraging, but there is still significant room for improvement.

### Decoding data from multiple peaks using logistic regression

Assuming that the discriminating peaks are partially uncorrelated (Fig D in S1 File), it is reasonable to seek improvement by utilizing multiple m/z peaks per metabolite. Such strategies will become increasingly important in more complex metabolomes.

**Fig 5:**
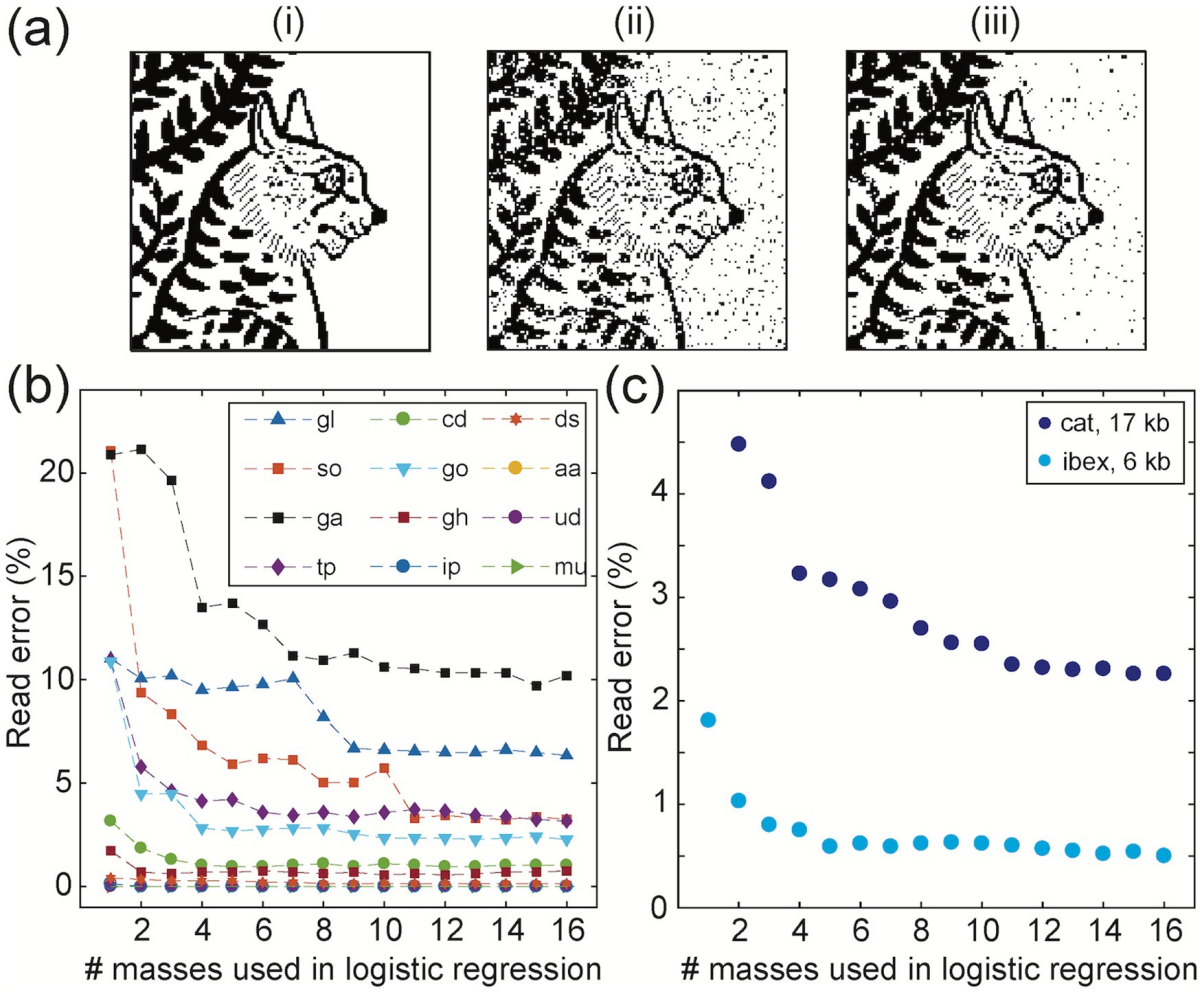
Logistic regression for multi-peak molecular data readout. Improvements over single-peak classification can be achieved with logistic regression utilizing multiple identifying masses per metabolite. **(a)** (i) A 17,424-bit image written into 1452 mixtures from a 12-metabolite subset of the library. (ii) The image recovered using one discriminating mass per metabolite. (iii) The image recovered using a regression combining 16 peaks per molecule. **(b)** Some compounds achieve low error rates even with single peaks. However, other molecules do not have an isolated m/z peak that shows high accuracy by itself. For these compounds, multi-peak logistic regression improves classification. **(c)** Cumulative read error rates for the two images as a function of the number of masses used in the logistic regression.

Using techniques similar to those for the 6kb ibex image, we encoded a 17,424-bit image of a cat from an Egyptian tomb [31] using 1,452 spots containing data mixtures from a 12-metabolite subset of the library (Fig 5a). We used this data to extend the decoding scheme to incorporate multiple m/z features. After identifying the set of statistically discriminating peaks, we performed a logistic regression using between 1 and 16 of the best-performing peaks. Multi-mass regression achieved a read accuracy of 97.7% for the whole cat image (Fig 5c). Cumulative read error rates for the data in Figs 4 and 5 are shown as a function of the number of masses used in the logistic regression. Applying these techniques to the earlier ibex dataset, an error rate of <0.5% was achieved. However, repeated measurement of spots can cause data loss. It was found that <1% error was added by each successive read of a data plate (Fig E in S1 File). Using a different plate for training achieved the same accuracy without overfitting (Fig F in S1 File). Overall, these demonstrations show that the metabolome is a viable and robust medium for representing digital information.

## Discussion

One advantage of molecular data storage is its high storage density. To date, demonstrations using DNA have reached about 214 petabytes per gram [32], although this is still orders of magnitude from theoretical limits [33]. For moderate amounts of data, an encoded metabolome written using a large small-molecule library could improve on this number [34], thanks to its increased chemical diversity. Our experiments highlight several limitations and potential benefits that warrant further discussion.

Statistically discriminating m/z features were used to classify the metabolite mixtures and recover the data at 98-99.5% accuracy using a simple analysis. Further development can take advantage of the wide range of sophisticated analysis technologies for metabolic profiling, including artificial neural networks, genetic algorithms, and self-organizing maps [35]. The inclusion of these methods, in conjunction with error correcting codes, leaves ample headroom for improved data recovery from more complex mixtures.

In terms of data rates, we demonstrated write speeds of 5 bits/sec, and aggregate read speeds of 11 bits/sec. We have performed little optimization of either the read or write times, and as the size of the metabolite library is increased, the MS read speed in particular has significant room to improve.

Looking forward, it would be interesting to consider the upper bound on information capacity using all known metabolites (∼ 10^5^ [14]). Even if only a fraction are stable, detectable, and display unique masses, this conservatively predicts hundreds of bits per spectral acquisition, which could all be read in parallel. As sub-zeptomole MS and nanomolar concentration detection have been available for nearly two decades [36, 37], detection at this level of complexity seems plausible.

Improvements in spatial density, and perhaps write speed, could come from reducing the volume and pitch of spots. There are opportunities for high density multilayer printing. To avoid storage density limits arising from finite transfer volumes, the precise mixture of metabolites associated with one spot can be pre-mixed in one well of an intermediary data plate. Transfer of 2.5 nL from the intermediary plate well to one spot means that hundreds of metabolites can be present in a nL volume on the plate. There is also room to extend on this work using larger libraries for higher capacity, or by storing multiple bits per complex, leveraging oligomerization [38].

In terms of density, we elected to use millimeter-scale arrays compatible with commercial instrumentation. Scaling the mixture spots down to diffraction-limited laser spot scales could improve data storage density by 6 orders of magnitude. Theoretically, this could facilitate extension from kilobyte- to gigabyte-scale data sets per plate. However, the true limit of data storage density depends on the available instrumentation.

ICR-MS (or other high-resolution MS such as orbital traps) have a finite ion capacity per acquisition, so the number of compounds can not be arbitrarily increased due to competition. Metabolites with a lower ionization efficiency will be excluded even though present in a large, competitive mixture. Therefore, to increase the number of metabolites per spot, future work may need to screen libraries for ionization efficiency. Alternatively, other read strategies (e.g. nanopores [39–41]) could provide higher sensitivity.

A likely source of error in more complex mixtures will be interactions between metabolites [5]. However, interspecies networks may also have benefits, such as opportunities for overwriting or transforming data, which hints at possibilities for synthetic metabolomic computation. One recurring challenge in metabolomics is obtaining trustworthy ‘ground truth’ samples. Perhaps by considering metabolomes as more abstract and mutable stores of information, we can develop new tools that allow us to overcome statistical biases, establish ground truths, and tease out subtle interactions and interconversion rates in well-regulated synthetic metabolomes.

## Conclusion

‘Omics’ technologies have grown out of genomics to encompass other complex information-rich systems like the metabolome. It is natural to ask whether there exist complementary opportunities to make use of metabolites’ structural diversity and interactivity. As a proof of principle of postgenomic small-molecule information storage, we have experimentally encoded >100,000 bits of digital images into synthetic metabolomes (Table B in S1 File), and we are confident that this number can be increased significantly in the future. One novel contribution is the demonstration of data storage in a mixture of dissimilar molecules, which can improve information capacity and read times through diversity and parallelism. Perhaps more importantly, this work offers a new perspective on small-molecule chemical information, and it introduces possibilities for synthetic metabolomic computation and establishing metabolic ‘ground truths’ through interrogation of synthetic metabolomes.

## Supporting information

S1 File Supporting information. Additional details about library compounds, read error rates, dataset sizes, repeated reads, data plates, error correlations, training cross-validation, and adducts.
(PDF)

## References

[1] Kell D. B. & Oliver S. G. The metabolome 18 years on: a concept comes of age. *Metabolomics*. 12(9), 148 (2016). 10.1007/s11306-016-1108-4 27695392 PMC5009154

[2] Manzoni C. Kia D. A. Vandrovcova J. Hardy J. Wood N. W. Lewis P.A. Genome, transcriptome and proteome: the rise of omics data and their integration in biomedical sciences. *Briefings in Bioinformatics*. 19(2), 286–302 (2016). 10.1093/bib/bbw114 PMC6018996 27881428

[3] Schmölz L. Birringerm M, Lorkowski S. & Wallert M. Complexity of vitamin E metabolism. *World J. Biol Chem*. 7(1), 14–43 (2016). 10.4331/wjbc.v7.i1.14 26981194 PMC4768118

[4] Meiser J. Weindl D. & Hiller K. Complexity of dopamine metabolism. *Cell Comm. and Siga*. 11:34 (2013). 10.1186/1478-811X-11-34 PMC3693914 23683503

[5] Sung J. Kim S. Jill Taar Cabatbat J. Jang S. Jin Y. Jung G. Y. et al Global metabolic interaction network of the human gut microbiota for context-specific community-scale analysis. *Nature Communications*. 8, 15393 (2017). 10.1038/ncomms15393 PMC5467172 28585563

[6] Dettmer K. Aronov P. A. & Hammock B.D. Mass spectrometry-based metabolomics. *Mass Spectrom Rev*. 26(1), 51–78 (2007). 10.1002/mas.20108 16921475 PMC1904337

[7] Zhang A. Sun H. Yan G. Wang P. & Wang X. Mass spectrometry-based metabolomics: applications to biomarker and metabolic pathway research. *Biomed Chromatogr*. 30(1), 7–12 (2016). 10.1002/bmc.3453 25739660

[8] Park B. K. Boobis A. Clarke S. Goldring C. E. Jones D. Kenna J. G. et al Managing the challenge of chemically reactive metabolites in drug development *Nature Reviews Drug Discovery*. 10, 292–306 (2011). 10.1038/nrd3408 21455238

[9] Sumner L. W. Lei Z. Nikolaubc B. J. & Saitode K. Modern plant metabolomics: advanced natural product gene discoveries, improved technologies, and future prospects. *Nat. Prod. Rep*. 32(2), 212–229 (2015). 10.1039/c4np00072b 25342293

[10] Gowda G. A. & Djukovic D. Overview of mass spectrometry-based metabolomics: opportunities and challenges. *Methods Mol Biol*. 1198, 3–12 (2014). 10.1007/978-1-4939-1258-2_1 25270919 PMC4336784

[11] Matsuda F. Technical Challenges in Mass Spectrometry-Based Metabolomics. *Mass Spectrom (Tokyo)* 5(2), S0052 (2016). 10.5702/massspectrometry.S0052 27900235 PMC5121192

[12] Zampieri M. Sekar K. Zamboni N. & Sauer U. Frontiers of high-throughput metabolomics. *Current Opinion in Chem. Bio*. 36, 15–23 (2017). 10.1016/j.cbpa.2016.12.006 28064089

[13] Brown M. Dunn W. B. Dobson P. Patel Y. Winder C. L. Francis-McIntyre S. et al Mass spectrometry tools and metabolite-specific databases for molecular identification in metabolomics. *Analyst*. 134(7), 1322–32 (2009). 10.1039/b901179j 19562197

[14] Wishart D. S. Feunang Y. D. Marcu A. Guo A. C. Liang K. Vázquez-Fresno R. et al HMDB 4.0: the human metabolome database for 2018. *Nucleic Acids Res*. 4(46), 608–617 (2018) 10.1093/nar/gkx1089 PMC5753273 29140435

[15] Davis J. Microvenus. *Art J*. 55, 70 (1996). 10.2307/777811

[16] Church G. M. Gao Y. & Kosuri S. Next-Generation Digital Information Storage in DNA. *Science*. 337(6102), 1628 (2012). 22903519 10.1126/science.1226355

[17] De Silva P. Y. & Ganegoda G. U. New Trends of Digital Data Storage in DNA. *Biomed. Res. Int*. 8072463 (2016). 10.1155/2016/8072463 27689089 PMC5027317

[18] Cox J. & Mann M. Quantitative, High-Resolution Proteomics for Data-Driven Systems Biology. *Ann. Rev. of Biochem*. 80, 273–299 (2011). 10.1146/annurev-biochem-061308-093216 21548781

[19] Nagaraj N. Wisniewski J. R. Geiger T. Cox J. Kircher M. Kelso J. et al Deep proteome and transcriptome mapping of a human cancer cell line. *Molecular Systems Biology* 7(1), 548 (2011). 10.1038/msb.2011.81 22068331 PMC3261714

[20] Roumpeka D. D. Wallace R. J. Escalettes F. Fotheringham I. & Watson M. A Review of Bioinformatics Tools for Bio-Prospecting from Metagenomic Sequence Data. *Front. Genet*. 8, 23 (2017). 10.3389/fgene.2017.00023 28321234 PMC5337752

[21] Hiller K. Hangebrauk J. Jäger C. Spura J. Schreiber K. & Schomburg D. MetaboliteDetector: comprehensive analysis tool for targeted and nontargeted GC/MS based metabolome analysis. *Anal Chem*. 81(9) 3429–39 (2009). 10.1021/ac802689c 19358599

[22] Peregrín-Alvarez J. M. Sanforf C. Parkinson J. The conservation and evolutionary modularity of metabolism. *Genome Biol*. 10(6): R63 (2009). 10.1186/gb-2009-10-6-r63 19523219 PMC2718497

[23] Cartwright J. H. E., Giannerini S. & Gonzalez D. L. DNA as information: at the crossroads between biology, mathematics, physics and chemistry. Phil. *Trans. Roy. Soc. of London A: M., Phys. and Eng. Sci*. 374 (2064), (2016). 10.1098/rsta.2015.0071 PMC4760126 26857674

[24] Wei P. Li B. Leon A.D. & Pentzer E. Beyond binary: optical data storage with 0, 1, 2, and 3 in polymer films. *J. Mat. Chem. C*. 5(23), 5780–5786 (2017). 10.1039/C7TC00929A

[25] Green J. E. Choi J. W. Boukai A. Bunimovich Y. Johnston-Halperin E. DeIonno E. et al A 160-kilobit molecular electronic memory patterned at 1011 bits per square centimetre. *Nature*. 445, 414, (2007). 10.1038/nature05462 17251976

[26] Sarkar T. Selvakumar K. Motiei L. & Margulies D. Message in a molecule. *Nat. Comm*. 7, 11374 (2016). 10.1038/ncomms11374 PMC4857388 27138465

[27] Arcadia, C. E. Hokchhay, T. Dombroski, A. Ferguson, K. Chen, S. L. Kim, E. et al. Parallelized Linear Classification with Volumetric Chemical Perceptrons. *IEEE International Conference on Rebooting Computing (ICRC)* (2018).

[28] The Rhode Island Hope Regiment Colors 1781. Rhode Island State House, 82 Smith St. Providence, RI. USA.

[29] Nikolaev EN. Kostyukevich YI. & Vladimirov GN. Fourier transform ion cyclotron resonance (FT ICR) mass spectrometry: Theory and simulations. *Mass Spectrom Rev*. 35(2), 219–258 (2016). 10.1002/mas.21422 24515872

[30] Unknown artist. ‘Ibex or Gazelle, Block Print’, 13th or 14th century Egyptian. Ink and white pigment on paper. Accession 2016.624. Gallery 454. Metropolitan Museum of Art. Fifth Avenue, NY. USA.

[31] Wilkinson, C. K. ‘Cat Killing a Serpent’, 1921. Facsimile made with Tempera on paper. Accession 30.4.1. Gallery 135. Metropolitan Museum of Art, Fifth Avenue, NY. USA.

[32] Erlich Y. & Zielinski D. DNA Fountain enables a robust and efficient storage architecture. *Science*. 355, 950–954 (2017). 10.1126/science.aaj2038 28254941

[33] Rose C. & Wright G. Inscribed matter as an energy-efficient means of communication with an extraterrestrial civilization. *Nature* 431, 47–49 (2004). 10.1038/nature02884 15343327

[34] Rosenstein, J. K. Rose, C. Reda, S. Weber, P. M. Kim, E. Sello, J. et al. Principles of Information Storage in Molecular Mixtures, arXiv:1905.02187, *submitted*.

[35] Kouskoumvekaki I. & Panagiotou G. Navigating the Human Metabolome for Biomarker Identification and Design of Pharmaceutical Molecules. *J Biomed Biotechnol*. 525497 (2011). 10.1155/2011/525497 20936122 PMC2948926

[36] Tang Y. Pingitore F. Mukhopadhyay A. Phan R. Hazen T. C. & Keasling J. D. Pathway Confirmation and Flux Analysis of Central Metabolic Pathways in Desulfovibrio vulgaris Hildenborough using Gas Chromatography-Mass Spectrometry and Fourier Transform-Ion Cyclotron Resonance Mass Spectrometry. *J. Bact*. 189, 940–949 (2007). 10.1128/JB.00948-06 17114264 PMC1797301

[37] Belov M. E. Gorshkov M. V. Udseth H. R. Anderson G. A. & Smith R. D. Zeptomole-Sensitivity Electrospray Ionization Fourier Transform Ion Cyclotron Resonance Mass Spectrometry of Proteins. *Anal. Chem*. 72(10), 2271–2279 (2000). 10.1021/ac991360b 10845374

[38] Martens S. Landuyt A. Espeel P. Devreese B. Dawyndt P. & Du Prez F. Multifunctional sequence-defined macromolecules for chemical data storage. *Nat. Commun*. 9, 4451 (2018). 10.1038/s41467-018-06926-3 30367037 PMC6203848

[39] Kennedy E. Dong Z. Tennant C. & Timp G. Reading the primary structure of a protein with 0.07 nm^3^ resolution using a subnanometre-diameter pore. *Nat. Nano*. 11(11), 968 (2016). 10.1038/nnano.2016.120 27454878

[40] Arcadia C. E., Reyes C. C., & Rosenstein J.K. In Situ Nanopore Fabrication and Single-Molecule Sensing with Microscale Liquid Contacts. *ACS Nano*, 11 (5), pp. 4907–4915, 2017 10.1021/acsnano.7b01519 28485922

[41] Galenkamp N.S. Soskine M. Hermans J. Wloka C. & Maglia G. Direct electrical quantification of glucose and asparagine from bodily fluids using nanopores. *Nat. Commun*. 9, 4085 (2018). 10.1038/s41467-018-06534-1 30291230 PMC6173770

