# Supplementary material for: Encoding information in synthetic metabolomes
Source: PLoS One. 2019 Jul 3;14(7):e0217364. doi: 10.1371/journal.pone.0217364 (PMC6608926; doi:10.1371/journal.pone.0217364)
Supplement: S1 File — Additional details about library compounds, read error rates, dataset sizes, repeated reads, data plates, error correlations, training cross-validation, and adducts. (PDF) [file pone.0217364.s001.pdf]

Supplementary Information for

Encoding Information in Synthetic Metabolomes

Eamonn Kennedy, Christopher E. Arcadia, Joseph Geiser, Peter M. Weber, Christopher Rose, Brenda M. Rubenstein, and Jacob K. Rosenstein

May 31, 2019

Synthetic metabolome library

Key	Name	Description	CID	Mass
**aa**	cis-Aconitic acid	acid	643757	174.016
**cm**	Creatine monohydrate	ADP to ATP recycler	80116	149.08
**es**	Sodium ethyl sulfate	alcohol metabolite	23680278	147.981
**gm**	Glutamine	amino acid	738	146.069
**mt**	Methionine	amino acid	6137	149.051
**hd**	Histidine	amino acid	6274	155.069
**pa**	Phenylalanine	amino acid	6140	165.079
**la**	L-(+)-Arginine	amino acid	6322	174.112
**ts**	Tyrosine	amino acid	6057	181.074
**tp**	Tryptophan	amino acid	6305	204.09
**ga**	N-Acetyl-L-glutamic acid	arginine intermediate	70914	189.064
**dr**	D-(-)-Ribose	carbohydrate	5311110	150.053
**ip**	Imidazolepropionic acid	histidine metabolite	70630	140.059
**pp**	4-Hydroxyphenylpyruvic acid	keto acid	979	180.042
**ad**	Adenosine 5'-diphosphate sodium	energy recycler	6022	427.029
**gl**	D-(+)-Galactose	monosaccharide	6036	180.063
**td**	Thymidine	nucleoside	5789	242.09
**cd**	Cytidine	nucleoside	6175	243.086
**ud**	Uridine	nucleoside	6029	244.07
**da**	2'-Deoxyadenosine	nucleoside	13730	251.102
**mu**	5-Methyluridine	nucleoside	445408	258.085
**as**	Adenosine	nucleoside	60961	267.097
**dm**	2'-Deoxyadenosine monohydrate	nucleoside	9549172	269.112
**go**	Guanosine	nucleoside	6802	283.092
**gh**	2'-Deoxyguanosine hydrate	nucleoside	16218597	285.107
**ct**	Cytidine 5'-triphosphate NaCl	nucleoside	16219171	526.948
**gp**	D-Glucose 6-phosphate sodium salt	pathway initiator	23702133	282.012
**na**	NADP Disodium	pathway initiator	2734411	787.039
**dc**	2'-Deoxycytidine	pyrimidine metab.	13711	227.091
**du**	2'-Deoxyuridine	pyrimidine metab.	13712	228.075
**ds**	Disodium succinate	salted acid	9020	161.99
**sc**	Trisodium Citrate Dihydrate	salted acid	71474	293.994
**so**	Sorbitol	sugar alcohol	5780	182.079
**th**	Thiamine hydrochloride	vitamin	6202	336.058
**rf**	Roboflavin	vitamin	493570	376.138
**pq**	Phylloquinone	vitamin	5284607	450.35

Table A: A list of screened compounds. The shown mass is the monoisotopic mass, as found on PubChem [1].

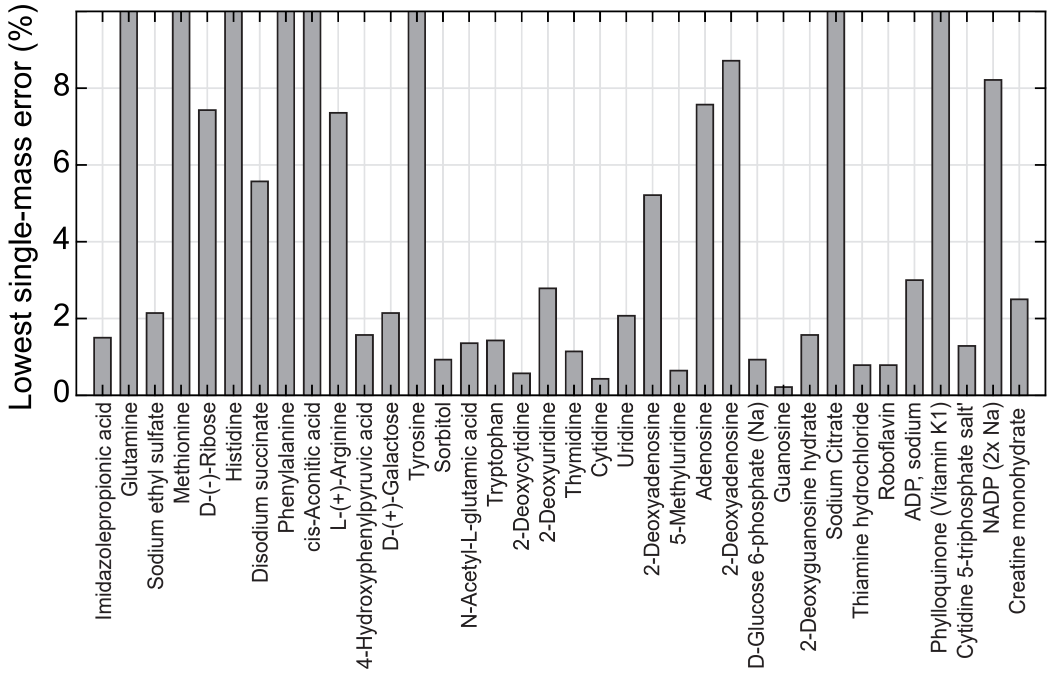

Figure A: Read error rates for each component of the synthetic metabolome. The data is derived from a 1400-spot plate, where each metabolite was prescribed pseudo-randomly as present or absent. Since each spot contained a mixture of 3 present and 33 absent metabolites, the error rates shown consider a degree of mixture error due to metabolic conversion. 8 / 36 metabolites have single-best-peak error rates > 10%, possibly due to poor uptake and solvation in DMSO. About half of the compounds yielded single-best-peak error rates of < 2%.

**Description**	**Ref.**	**bits**	**repetitions**	**net kbits**
Ibex print	[2]	6,142	2×	12.3
RI Flag 1781	[3]	8,904	3×	26.7
Cat drawing	[4]	17,424	4×	69.7

Table B: A list of image data sets written, with the number of repetitions. Cumulatively, ≈108,700 bits were written into synthetic metabolomes.

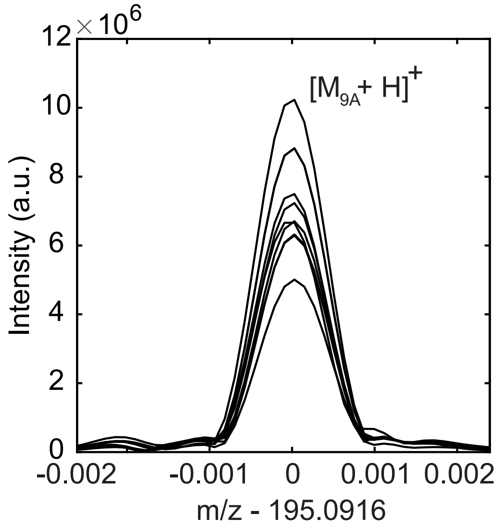

Figure B: Eight repetitions of MALDI mass spectral acquisition are shown centered at m/z = 195.0916 (protonated 9-Aminoacridine). Each repetition is from a unique deposition of 40 nL of 18.25 mM matrix in DMSO (air dried). The entirety of the peak above background is captured within the spectral window range M ± 0.001 m/z, regardless of signal intensity.

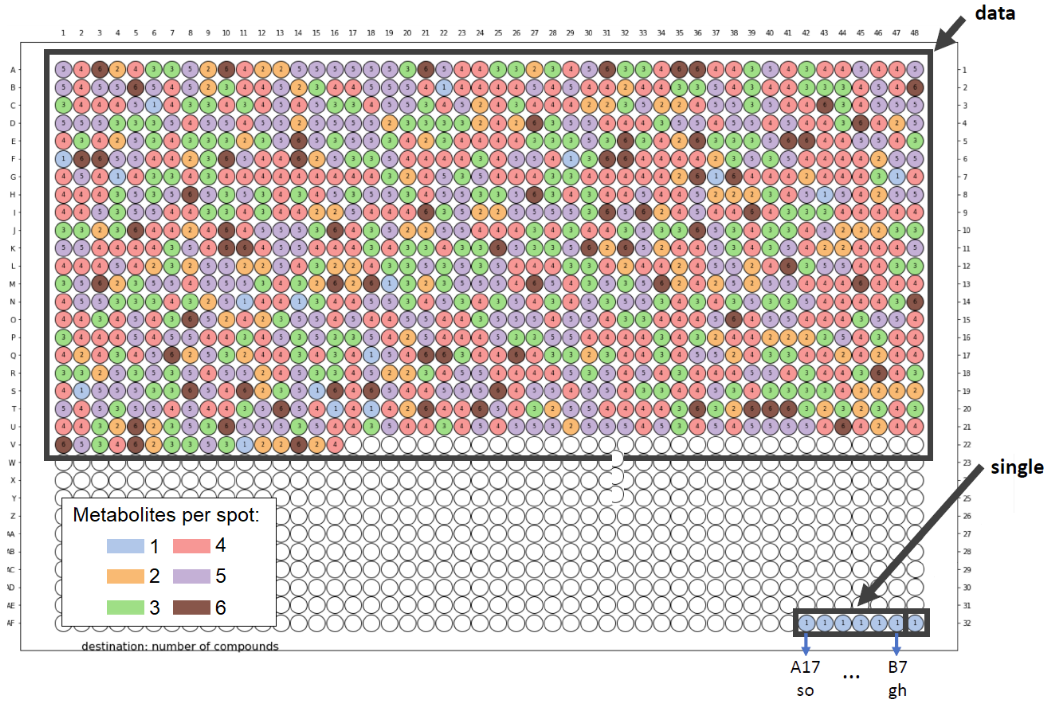

Figure C: (a) A graphical overview of the Nubian ibex data plate contents. A color-coding is used to denote the number of metabolites present in each of the 1024 spot mixtures.

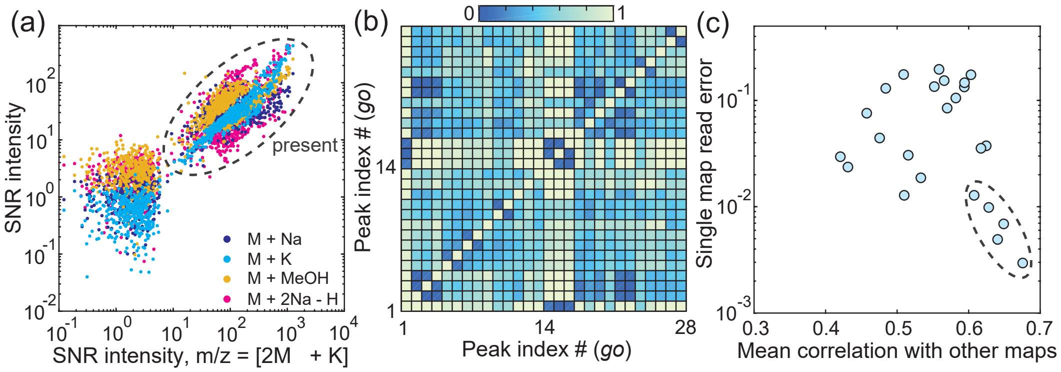

Figure D: Appreciating the degree of correlation in readout errors using multiple m/z peaks. Each mass is associated with a list of normalized intensities from each spot on a plate. (a) The normalized intensity of the $[2M+K]^+$ peak from all 1024 locations is shown for guanosine, plotted against the intensities of other guanosine ions. The intensities clustered into present (dotted ellipse) and absent states, and ion intensities are positively but imperfectly correlated. In (b), the 28 best discriminating masses are selected for autocorrelation. Some sets of masses exhibit clustered groupings, but correlations are imperfect. (c) The effective read error at each m/z is plotted against its mean correlation with other guanosine features. The masses which yield the lowest errors are often more correlated (dotted ellipse).

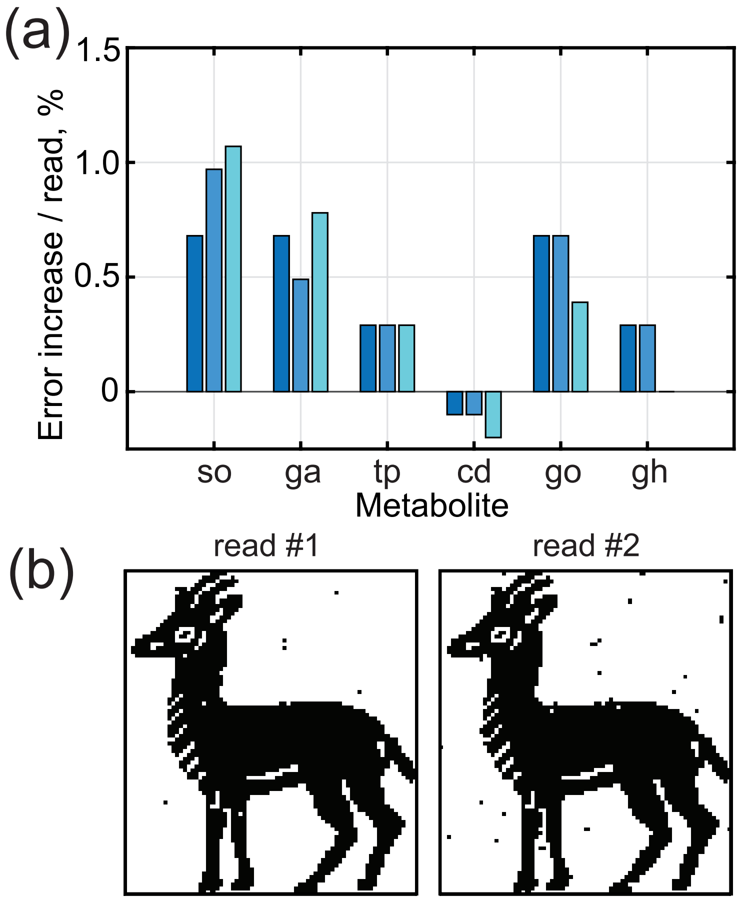

Figure E: Repeated measurement of spots can cause data loss. A 6142 bit image data plate was written using 6 metabolites as described in Figure 4. The plate was read several times. The increase in error rate per read is shown in (a) broken out by metabolite. The first and second read repetition of the image using 16-peak logistic regression (see Methods) are shown in (b). Each read took <2 hrs. Typically, <1% error was added by each successive measurement of a data plate.

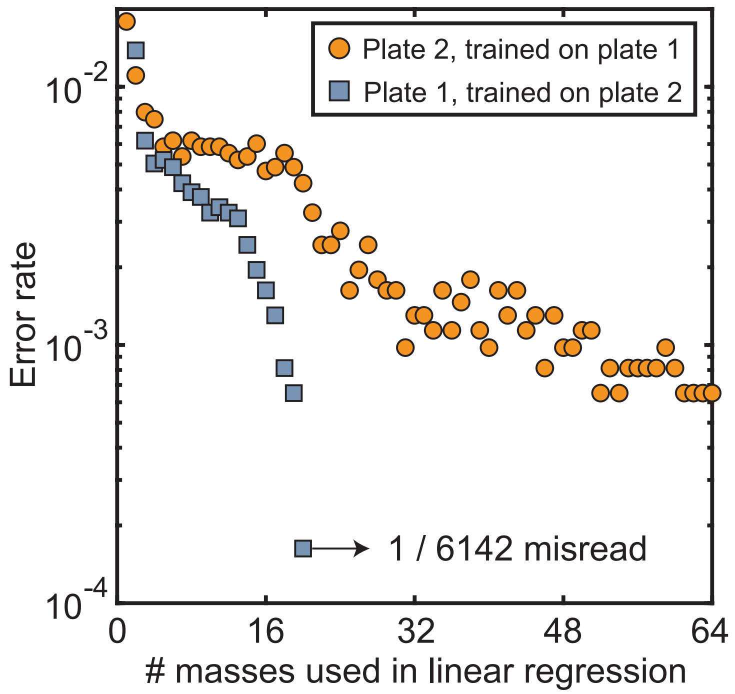

Figure F: Cross validation. Two identical 6142 bit image data plates, plate 1 and plate 2, were both written using the 6 metabolites described in Figure 4. Plate 1 was used to train the logistic regression to determine which masses were optimal discriminators. These masses were then used to recover data from plate 2. The process was then reversed. Error rates are shown as a function of the number of identifying masses used in regression, where training and testing of the data has been seperated as described. Although some plate-specific complexity is evident, aggregate error rates dropped below 0.1% in both cases, with complete seperation of training and test data.

Name	M_{obs} - M_{mono}	Adduct	Accuracy (%)
**Guanosine**	22.99	Na	99.786
**Cytidine**	38.96	K	99.571
**2'-Deoxycytidine**	22.99	Na	99.429
**5-Methyluridine**	22.99	Na	99.357
**Thiamine**	2.015	^{2}H	99.214
**Roboflavin**	22.99	Na	99.210
**Sorbitol**	30.97	P	99.071
**D-Glucose 6-phosphate**	22.99	Na	99.070
**Thymidine**	22.99	Na	98.857

Table C: Adduct classification and data recovery accuracy. The best discrminating peak mass minus the monoisotoic mass (M_{obs} - M_{mono}) is shown for metabolites. The adduct type is determined from the residual mass. The accuracy recovered from each of the adducts is shown.

Multiple (n)	M_{obs} - n$\times M_{mono}$	Adduct	Accuracy (%)
**1**	22.99	Na	99.786
**1**	23.99	Na + 1_c	98.926
**1**	38.96	K	99.604
**1**	44.97	2Na - H	98.729
**2**	22.99	Na	95.2150
**2**	38.95	K	94.7270
**2**	23.99	Na + 1_c	94.0430
**2**	30.97	P	91.1300
**2**	44.96	2Na - H	90.7230
**2**	39.95	K + 1_c	88.6700

Table D: Guanosine adduct list. The discrminating peak mass minus the monoisoptic mass (M_{obs} - M_{mono}) is shown for 10 peaks associated with Guanosine. The adduct type is determined from the residual mass. The data recovery accuracy for each mass multiple and adduct is shown. For large absolute m/z, the error in m/z increases due to finite sampling limitations.

References

[1] Kim, S. Thiessen, P. A. Bolton, E. E. Chen, J. Fu, G. Gindulyte, A. et al. PubChem Substance and Compound databases. *Nucleic Acids Res.* 44, 1202 (2016).

[2] Unknown artist. ‘Ibex or Gazelle, Block Print’, 13th or 14th century Egyptian. Ink and white pigment on paper. Accession 2016.624. Gallery 454. Metropolitan Museum of Art. Fifth Avenue, NY. USA.

[3] The Rhode Island Hope Regiment Colors 1781. Rhode Island State House, 82 Smith St. Providence, RI. USA.

[4] Wilkinson, C. K. ‘Cat Killing a Serpent’, 1921. Facsimile made with Tempera on paper. Accession 30.4.1. Gallery 135. Metropolitan Museum of Art, Fifth Avenue, NY. USA.
